# Relationship Between COVID-19 Threat Beliefs and Individual Differences in Demographics, Personality, and Related Beliefs

**DOI:** 10.3389/fpsyg.2022.831199

**Published:** 2022-02-18

**Authors:** Ana Butkovic, Mirta Galesic

**Affiliations:** ^1^Department of Psychology, Faculty of Humanities and Social Sciences, University of Zagreb, Zagreb, Croatia; ^2^Santa Fe Institute, Santa Fe, NM, United States; ^3^Harding Center for Risk Literacy, University of Potsdam, Potsdam, Germany

**Keywords:** COVID-19 threat beliefs, individual differences, personality, trust, vaccination

## Abstract

Individual differences in demographics, personality, and other related beliefs are associated with coronavirus disease 2019 (COVID-19) threat beliefs. However, the relative contributions of these different types of individual differences to COVID-19 threat beliefs are not known. In this study, a total of 1,700 participants in Croatia (68% female; age 18–86 years) completed a survey that included questions about COVID-19 risks, questions about related beliefs including vaccination beliefs, trust in the health system, trust in scientists, and trust in the political system, the HEXACO 60 personality inventory, as well as demographic questions about gender, age, chronic diseases, and region. We used hierarchical regression analyses to examine the proportion of variance explained by demographics, personality, and other related beliefs. All three types of individual differences explained a part of the variance of COVID-19 threat beliefs, with related beliefs explaining the largest part. Personality facets explained a slightly larger amount of variance than personality factors. These results have implications for communication about COVID-19.

## Introduction

Understanding individual differences that contribute to coronavirus disease 2019 (COVID-19) threat beliefs is important for better management of the disease (Bruine de Bruin and Bennett, 2020; Astley et al., 2021). For example, gender differences in both beliefs and compliance with rules were found in Australia, Austria, France, Germany, Italy, New Zealand, the United Kingdom, and the United States (Galasso et al., 2020). Knowledge about individual differences can help tailor specific messages for different parts of the population that needs to be reached to minimize the spreading of COVID-19.

In this study, we investigated how beliefs about COVID-19 risks are associated with the following three different types of individual differences: (i) differences in demographic characteristics such as gender, age, health status, and region, (ii) differences in personality traits, and (iii) differences in other related beliefs such as trust in doctors, science, and the government. As we reviewed next, each of these types of individual differences explains some of the variances in COVID-19 threat beliefs. However, to the best of our knowledge, no study so far has examined the relative contributions of all of these variables in a joint study. This is important because knowing the relative contribution of different types of individual differences can help focus educational efforts to obtain the most leverage in changing beliefs about COVID-19 risks.

### Demographic Characteristics

Gender and age are important factors for the prognosis of COVID-19 infection, with more women getting infected but more men dying and with the majority of deaths occurring in the more than 65 years age group (WHO, 2021). Therefore, gender and age differences in COVID-19 threat beliefs have been examined worldwide. Studies have found that women and older adults perceive and believe COVID-19 is a more serious threat and are more afraid of it (Buljan Flander et al., 2020; Dryhurst et al., 2020; Galasso et al., 2020; Gerhold, 2020; Szabo et al., 2020; Alsharawy et al., 2021; Bechard et al., 2021; Laires et al., 2021; Rana et al., 2021). Another important factor influencing COVID-19 threat beliefs is a chronic disease diagnosis, since people with chronic conditions can be more likely to become severely ill from COVID-19 (CDC, 2021). Studies in Portugal (Laires et al., 2021) and the United States (Ricotta et al., 2021) have shown that people with chronic diseases are more likely to believe that COVID-19 is a threat. Finally, some studies have examined and found regional differences in COVID-19 threat beliefs, for example, in Germany (Gerhold, 2020) and Canada (Parsons Leigh et al., 2020). In Croatia, historical trajectories and contemporary societal developments led to cultural differences between regions. These are reflected, to an extent, in cultural dimensions as measured using Hofstede’s approach (Rajh et al., 2016), which could be related to regional differences in COVID-19 threat beliefs. Hofstede’s model assumes that the most important cultural issues can be captured by the following six cultural dimensions: Power Distance, Uncertainty Avoidance, Individualism/Collectivism, Masculinity/Femininity, Long/Short Term Orientation, and Indulgence/Restraint (Hofstede, 2011). Gokmen et al. (2021) hypothesized that Individualism, Masculinity, and Indulgence would have a positive effect, while Power Distance, Uncertainty Avoidance, and Long-Term Orientation would have a negative effect on the rate of increase in COVID-19 cases in selected European countries. They found support for their hypotheses for Power Distance, Individualism, and Indulgence.

### Personality Traits

Personality traits influence our interactions with and modifications to the psychological, social, and physical environments (Larsen and Buss, 2017), so studies have examined how personality traits are associated with COVID-19 risk perceptions and behavioral adaptations to the pandemic. Most studies used the Big Five personality framework. A prospective panel study that collected personality data prior to the pandemic showed that personality explained only a small portion of outcome measures, with personality facets explaining more variance than personality factors (Rammstedt et al., 2021). Aschwanden et al. (2021) assessed personality at one time point and then, approximately a month later, psychological and behavioral responses to COVID-19. Results indicated that personality traits, specifically neuroticism, extraversion, and conscientiousness, predicted relevant psychological and behavioral responses to COVID-19. In addition, specific personality facets from the lower hierarchy level had more predictive power than personality traits. Schmiedeberg and Thönnissen (2021) found that neuroticism was associated with negative and openness to experience with positive perceptions of the COVID-19 pandemic. Tagini et al. (2021) found that higher openness was associated with a lower perception of risk. Several studies used the HEXACO personality framework to assess the relationship between personality and COVID-19 criteria. Zettler et al. (2022) reported that Emotionality/Neuroticism was the only consistent predictor of risk perception, while Honesty-Humility, Agreeableness, and Conscientiousness showed significant associations with behavioral adjustment. Lazarević et al. (2021) assessed Big Five factors together with Honesty-Humility and found that Honesty-Humility had the strongest association with recommended health practices. Therefore, it seems that using HEXACO personality framework to investigate the associations between personality and COVID-19-related outcomes can reveal interesting findings beyond those uncovered using the Big Five framework.

The relationship between personality and COVID-19-related impacts, concerns, and behaviors has been examined in different cultures (Al-Omiri et al., 2021). In general, personality was associated with COVID-19-related impacts, concerns, and behaviors, but these associations were somewhat different for participants from different countries. Therefore, it is important to examine these associations in different populations, including in Croatia where they have not been investigated so far.

### Related Beliefs

Beliefs about COVID-19 do not develop in a vacuum, but in the context of various other related beliefs. For example, people with higher COVID-19 risk perception are more likely to engage in preventative health behaviors (e.g., washing hands, wearing a face mask, and physical distancing; Dryhurst et al., 2020). When novel information about an issue contrasts already existing beliefs and values people have, cognitive dissonance can occur and impede acceptance of new facts (Festinger, 1954). Rather than modifying their existing beliefs to fit the new facts, people can be prone to motivated reasoning and interpret the new facts differently or disregard them in order to preserve their previous beliefs (Druckman and McGrath, 2019). In particular, people who distrust the health system (Udow-Phillips and Lantz, 2020; Antinyan et al., 2021), scientists (Bicchieri et al., 2021; Sturgis et al., 2021), and the overall political system (Bruine de Bruin et al., 2020; Schaeffer, 2021) can be more reluctant to believe the official messages about the risk of COVID-19 and less willing to adopt preventative measures such as vaccination. For example, in the United States, changes in political leadership and the related levels of trust in government messaging have been related to changes in beliefs about the risk of COVID-19 and willingness to accept vaccination. Many more Democrats have been supporting vaccination since the Democrat candidate won the 2020 election than before the election. Republicans, traditionally opposed to a stronger influence of government, have remained more skeptical than Democrats regarding both the COVID-19 risk and vaccination, similar to ideological right-leaning populations in most European countries (Connaughton, 2021).

### This Study

We examined how individual differences in demographics, personality, and other related beliefs contribute to COVID-19 threat beliefs. Based on previous findings (Buljan Flander et al., 2020; Dryhurst et al., 2020; Galasso et al., 2020; Gerhold, 2020; Szabo et al., 2020; Alsharawy et al., 2021; Bechard et al., 2021; Laires et al., 2021; Rana et al., 2021; Ricotta et al., 2021), we expected women, older people, and people with chronic diseases to believe that COVID-19 poses more risk, and we expected regional differences in beliefs about COVID-19 risks (Gerhold, 2020; Parsons Leigh et al., 2020). Furthermore, we expected that COVID-19 threat beliefs will be related to personality factors as operationalized using the HEXACO model (Lazarević et al., 2021; Zettler et al., 2022). Finally, we expected that COVID-19 threat beliefs will be associated with other related beliefs such as trust in the health system, scientists, and the political system (Bruine de Bruin et al., 2020; Udow-Phillips and Lantz, 2020; Antinyan et al., 2021; Bicchieri et al., 2021; Schaeffer, 2021; Sturgis et al., 2021).

## Methods

### Participants and Procedure

Psychology students were asked to nominate acquaintances with different demographic characteristics who were sent an email invitation for participation in the study. Data were collected from December 4 to 31, 2020 through the web questionnaire platform Unipark.^1^ A total of 1,700 participants completed the survey (68% female, age range: 18–86 years, *M* = 28.09, and SD = 12.50), coming from all 21 counties and representing the following four main Croatian regions (Croatian Bureau of Statistics, 2019): Pannonian Croatia (*N* = 179), Adriatic Croatia (*N* = 348), City of Zagreb (*N* = 852), and Northern Croatia (*N* = 321). Chronic disease was reported by 225 participants (13%). This study was approved by the Ethics Committee of the Department of Psychology, University of Zagreb.

### Measures

We measured four specific beliefs about COVID-19 risks (COVID-19 threat beliefs) using a 5-point Likert-type scale (1 = completely disagree; 5 = completely agree): COVID-19 *is less dangerous than the flu*, COVID-19 *is spreading rapidly*, COVID-19 *is dangerous only for old people*, and COVID-19 *measures help to stop the virus spreading*. Items measuring these and other related beliefs were selected in a pretest on a student sample (*N* = 105), including a larger set of items taken from other studies and generated by the authors. The selected items showed better psychometric properties including variability and item-total correlations. Items were recoded, and the total score was calculated so that the higher score indicated a higher belief that COVID-19 is a serious threat (Cronbach’s α = 0.67).

We measured personality traits using the HEXACO 60-item scale (Ashton and Lee, 2009) that includes the following subscales: Honesty-Humility, Emotionality, Extraversion, Agreeableness, Conscientiousness, and Openness to Experience. Participants had to indicate their agreement with items on a 5-point Likert-type scale. In line with other studies using Croatian translation (Babarović and Šverko, 2013), the internal-consistency reliabilities were satisfactory (Honesty-Humility α = 0.78; Emotionality α = 0.78; Extraversion α = 0.79; Agreeableness α = 0.74; Conscientiousness α = 0.68; Openness to Experience α = 0.77). Descriptive statistics and correlations for study variables are presented in Tables 1, 2, showing variability in COVID-19 threat beliefs and other individual differences. Internal consistency reliabilities for personality facets are shown in Table 2 and were also satisfactory.

**TABLE 1:** Descriptive statistics and correlations between the variables (*N* = 1,700).

	*M*	*SD*	(1)	(2)	(3)	(4)	(5)	(6)	(7)	(8)	(9)	(10)	(11)	(12)	(13)
(1) COVID 19	3.60	0.71													
(2) Gender	1.68	0.47	0.06												
(3) Age	28.09	12.50	0.14	0.04											
(4) Chronic condition	0.13	0.34	0.14	0.01	0.22										
(5) Honesty-Humility	3.51	0.65	0.12	0.15	0.27	0.06									
(6) Emotionality	3.38	0.63	0.10	0.47	−0.03	0.07	0.08								
(7) Extraversion	3.29	0.62	−0.03	−0.05	0.14	−0.03	0.01	−0.12							
(8) Agreeableness	3.05	0.58	0.08	0.00	0.04	−0.01	0.25	−0.08	0.06						
(9) Conscientiousness	3.51	0.57	0.14	0.06	0.14	0.05	0.19	0.07	0.15	0.02					
(10) Openness to experience	3.63	0.62	0.11	0.01	−0.01	0.01	0.02	−0.03	0.11	0.04	0.04				
(11) Vaccination	3.39	0.93	0.52	−0.13	0.01	0.09	−0.08	−0.04	−0.03	−0.01	0.09	0.13			
(12) Health system	3.03	0.85	0.28	0.00	0.06	−0.01	0.08	−0.01	0.11	0.14	0.07	−0.04	0.26		
(13) Scientists	3.93	0.74	0.49	−0.02	−0.02	0.07	0.01	0.01	0.00	0.07	0.14	0.19	0.54	0.24	
(14) Political system	2.79	0.78	0.29	0.01	−0.10	0.00	0.03	−0.01	0.06	0.13	0.07	−0.05	0.26	0.66	0.23

*All correlations ≥ ± 0.09 significant at p < 0.001; gender: 1 = males, 2 = females; chronic: 0 = no, 1 = yes; M = arithmetic mean.*

**TABLE 2:** Descriptive statistics for personality facets and correlations with coronavirus disease 2019 (COVID-19) threat beliefs.

Facet	*M*	*SD*	α	*r*
H: Sincerity	3.74	0.79	0.63	0.04
H: Fairness	3.65	1.04	0.75	0.15*
H: Greed avoidance	3.07	0.91	0.66	0.05
H: Modesty	3.36	0.85	0.48	0.06
E: Fearfulness	3.06	0.84	0.63	0.08
E: Anxiety	3.76	0.93	0.66	0.08
E: Dependence	3.18	0.99	0.73	0.04
E: Sentimentality	3.59	0.83	0.69	0.10*
X: Social self-esteem	3.39	0.86	0.71	−0.02
X: Social boldness	3.00	0.86	0.71	−0.05
X: Sociability	3.44	0.85	0.56	0.00
X: Liveliness	3.42	0.89	0.76	−0.02
A: Forgivingness	2.96	0.88	0.68	−0.02
A: Gentleness	3.20	0.76	0.56	0.04
A: Flexibility	2.77	0.72	0.45	0.09*
A: Patience	3.31	1.01	0.77	0.10*
C: Organization	3.35	0.94	0.52	0.07
C: Diligence	3.84	0.78	0.55	0.11*
C: Perfectionism	3.66	0.73	0.57	0.07
C: Prudence	3.24	0.75	0.55	0.14*
O: Aesthetic appreciation	3.70	0.93	0.61	0.13*
O: Inquisitiveness	3.49	0.93	0.47	0.13*
O: Creativity	3.53	0.92	0.74	0.03
O: Unconventionality	3.76	0.73	0.60	0.05

*p < 0.001. M = arithmetic mean; α = Cronbach’s alpha coefficient, r = correlation coefficient.

We measured the following four beliefs related to the different aspects of the societal situation during the COVID-19 pandemic: vaccination beliefs, trust in the health system, trust in scientists, and trust in the political system in Croatia, each with three items answered on a 5-point Likert-type scale (1 = completely disagree; 5 = completely agree). The vaccination conspiracy beliefs scale included the following (α = 0.77): *Vaccination in Croatia should be an obligation, not a matter of choice*; *Side effects of vaccination are milder than symptoms of different diseases*; and *Vaccination brings more harm than benefit*. The scale for trust in the health system included (α = 0.80): *I trust the health system in Croatia*, *If I was sick I am sure I would get the appropriate healthcare* and *In general, I think that the health system in my local community functions badly*. The scale for trust in scientists included the following (α = 0.75): *Scientists can offer valuable explanations for different phenomena*, *Scientists’ opinion is overvalued in our society*, and *Scientists care only about their discoveries*. The scale for trust in the political system included the following (α = 0.66): *Croatians can feel safe in crisis situations*, *Croatian citizens participate in making important political decisions*, and *Basic civil rights are guaranteed to Croatian residents*.

## Results

Descriptive statistics and correlations for study variables are presented in Tables 1, 2, showing variability in COVID-19 threat beliefs and other individual differences. Correlations between facets follow the overall structure of the correlations between factors shown in Table 1, with the only larger correlation being the one between the Extraversion facets, Social self-esteem, and Liveliness (*r* = 0.59, *p* < 0.001).

Older participants and those with a chronic condition had a higher belief that COVID-19 is a serious threat. COVID-19 threat beliefs correlated positively with Honesty-Humility and its facet Fairness, Emotionality and its facet Sentimentality, Conscientiousness and its facets Diligence and Prudence, and Openness to experience and its facets Aesthetic appreciation and Inquisitiveness. Although Agreeableness at the factor level was not correlated significantly with COVID-19 threat beliefs, its facets Flexibility and Patience showed positive correlations. Finally, COVID-19 threat beliefs correlated most strongly with all other related beliefs. To examine regional differences, we ran an ANOVA for COVID-19 threat beliefs with Tukey HSD comparisons [*F*_(3,1696)_ = 7.25, *p* < 0.001]. Significant differences were found between participants from the Adriatic region (*M* = 3.46 and SD = 0.75) and participants from the City of Zagreb (*M* = 3.66 and SD = 0.70).

Since our variables had significant intercorrelations, we further examined their associations with COVID-19 threat beliefs with hierarchical regression analyses. Power analysis for regression analysis indicated that our sample size is large enough to detect even a small effect size with 34 predictors with the power set at 0.90 and alpha level set at *p* < 0.05. We entered the demographic variables in the first step, personality factors or personality facets in the second step, and other beliefs in the third step. Since previous studies indicated that personality facets might explain more variance in COVID-19 threat beliefs than personality factors (Aschwanden et al., 2021; Rammstedt et al., 2021), we also examined the contribution of personality factors or personality facets in our analyses. The region was entered as dummy variables comparing three of the four regions to the Adriatic region, which had the lowest score for COVID-19 threat beliefs. None of the predictors had VIF larger than 1.1, indicating satisfactory levels of tolerance. In addition, plots of residuals against the predicted values suggested no deviations from normality and no evidence of heteroscedasticity.

As shown in Table 3, all three steps were significant for predicting COVID-19 threat beliefs with individual differences in other beliefs explaining the largest proportion of variance. Demographic variables accounted for 4.4% of the variability and personality factors contributed for an additional 4.3%, while personality facets explained an additional 7.7% of the variance in COVID-19 threat beliefs. In the final step, the Honesty-Humility facet, Fairness, was positively associated, and the Extraversion facet, Social boldness, was negatively associated with COVID-19 threat beliefs. Other beliefs explained most of the variance, with the full model accounting for 40% of the variance in COVID-19 threat beliefs. Vaccination beliefs, trust in scientists, and trust in the political system were significant predictors in both regression analyses.

**TABLE 3:** Summary of hierarchical regression analyses with standardized regression coefficients and SEs in parentheses.

	Step 1	Step 2	Step 3		Step 1	Step 2	Step 3
Gender	0.05 (0.024)	−0.01 (0.027)	0.04 (0.022)	Gender	0.05 (0.024)	0.00 (0.027)	0.04 (0.023)
Age	0.11* (0.024)	0.10* (0.025)	0.12* (0.021)	Age	0.11* (0.024)	0.09* (0.028)	0.13* (0.023)
Chronic condition	0.12* (0.024)	0.10* (0.024)	0.06* (0.02)	Chronic condition	0.12* (0.024)	0.10* (0.024)	0.06* (0.02)
Pannonian	0.04 (0.028)	0.04 (0.027)	0.04 (0.022)	Pannonian	0.04 (0.028)	0.05 (0.027)	0.05 (0.022)
Zagreb	0.14* (0.031)	0.14* (0.031)	0.06 (0.025)	Zagreb	0.14* (0.031)	0.15* (0.031)	0.08* (0.025)
Northern	0.09* (0.03)	0.08* (0.029)	0.04 (0.024)	Northern	0.09* (0.03)	0.10* (0.029)	0.05 (0.024)
Honesty-Humility		0.04 (0.026)	0.08* (0.021)	H: Sincerity		−0.02 (0.027)	0.02 (0.022)
Emotionality		0.09* (0.027)	0.09* (0.022)	H: Fairness		0.10* (0.028)	0.08* (0.023)
Extraversion		−0.07* (0.024)	−0.05 (0.02)	H: Greed avoidance		−0.03 (0.028)	−0.02 (0.023)
Agreeableness		0.07* (0.024)	0.03 (0.02)	H: Modesty		0.01 (0.026)	0.02 (0.022)
Conscientiousness		0.11* (0.024)	0.03 (0.02)	E: Fearfulness		0.06 (0.029)	0.01 (0.024)
Openness		0.11* (0.023)	0.03 (0.02)	E: Anxiety		0.04 (0.029)	0.04 (0.024)
Vaccination			0.34* (0.023)	E: Dependence		−0.02 (0.028)	−0.02 (0.023)
Health system			0.04 (0.026)	E: Sentimentality		0.05 (0.029)	0.06 (0.024)
Scientists			0.25* (0.023)	X: Social self-esteem		−0.05 (0.031)	−0.06 (0.026)
Political system			0.13* (0.026)	X: Social boldness		−0.05 (0.028)	−0.07* (0.023)
				X: Sociability		0.03 (0.028)	0.04 (0.023)
				X: Liveliness		0.01 (0.031)	0.03 (0.026)
				A: Forgivingness		−0.10* (0.027)	−0.06 (0.022)
				A: Gentleness		0.04 (0.028)	0.03 (0.023)
				A: Flexibility		0.06 (0.026)	0.02 (0.022)
				A: Patience		0.08* (0.027)	0.03 (0.022)
				C: Organization		−0.01 (0.028)	0.00 (0.023)
				C: Diligence		0.05 (0.028)	0.02 (0.023)
				C: Perfectionism		0.00 (0.027)	0.00 (0.022)
				C: Prudence		0.08* (0.029)	0.01 (0.024)
				O: Aesthetic appreciation		0.05 (0.028)	0.02 (0.023)
				O: Inquisitiveness		0.10* (0.026)	0.01 (0.021)
				O: Creativity		−0.01 (0.027)	0.02 (0.022)
				O: Unconventionality		0.05 (0.028)	0.00 (0.024)
				Vaccination			0.34* (0.024)
				Health system			0.03 (0.026)
				Scientists			0.25* (0.023)
				Political system			0.13* (0.026)
*F*	14.15* (6,1688)	14.07* (12,1682)	70.87* (16,1678)	*F*	14.15* (6,1688)	7.91* (12,1682)	34.64* (16,1678)
*Adj. R^2^*	0.044	0.085	0.398	*Adj. R^2^*	0.044	0.109	0.403
Δ *F*		13.38* (6,1682)	219.32* (4,1678)	Δ *F*		6.10* (6,1682)	205.85* (4,1678)
Δ *R*^2^		0.043	0.312	Δ *R*^2^		0.077	0.290

*p ≤ 0.01; Gender 1 = male, 2 = female. F = F-ratio; Adj. R^2^ = adjusted coefficient of determination; ΔF = change in F-ratios; ΔR^2^ = change in R^2^.

## Discussion

This study is, to our knowledge, the first to examine the relative contributions to COVID-19 threat beliefs of individual differences in demographics, personality, and other related beliefs. Our results both confirm some previous findings and add further novel findings to the literature.

Previous studies have indicated that gender (Buljan Flander et al., 2020; Dryhurst et al., 2020; Galasso et al., 2020; Gerhold, 2020; Szabo et al., 2020; Alsharawy et al., 2021; Rana et al., 2021), age (Buljan Flander et al., 2020; Galasso et al., 2020; Gerhold, 2020; Szabo et al., 2020; Bechard et al., 2021; Laires et al., 2021), chronic conditions (Laires et al., 2021; Ricotta et al., 2021), and region (Gerhold, 2020; Parsons Leigh et al., 2020) can explain some of the variability in different COVID-19 threat beliefs. In line with the previous findings, these variables were significant predictors of COVID-19 threat beliefs in the first step of our regression analyses, accounting for 4.4% of the variance, with age and chronic conditions remaining significant predictors in the last step. Older participants and those with a chronic condition believe that COVID-19 is a more serious threat. Although previous studies have indicated that women perceive COVID-19 as a more serious threat, in this study, gender was not significantly associated with COVID-19 threat beliefs. Our results echo the findings of Galasso et al. (2020), suggesting that gender differences in COVID-19 threat beliefs are not stable over time. In addition, we found regional differences in COVID-19 threat beliefs between the southern Adriatic region and the northern City of Zagreb, echoing results of a past study that found regional differences in Hofstede’s culture values, in particular the long-term orientation, between South and North Croatia (Rajh et al., 2016). These differences could be partially caused by the fact that the City of Zagreb is an urban region that is more densely populated than the Adriatic region, so the closer interpersonal proximity could have elevated the fear of COVID-19. However, the Adriatic region also had a lower level of COVID-19 threat beliefs when compared with the other two less urban regions, namely, the Northern and Pannonian regions, suggesting that the differences are also at least partially cultural.

Previous studies examining the association between personality and COVID-19 threat beliefs have mainly used a five-factor personality framework (Al-Omiri et al., 2021; Aschwanden et al., 2021; Rammstedt et al., 2021; Schmiedeberg and Thönnissen, 2021; Tagini et al., 2021) and have established that personality explains a significant, but a small amount of variance in COVID-19 threat beliefs, with personality facets explaining more variance than personality factors (Aschwanden et al., 2021; Rammstedt et al., 2021). Our findings were in line with previous findings, with personality factors accounting for a significant, but small, amount of variance in COVID-19 threat beliefs (4.3%) and personality facets accounting for more variance than personality factors in the second step of the regression analyses (7.7%). We used the HEXACO personality framework since the Honesty-Humility factor was an important personality predictor in past studies (Lazarević et al., 2021; Zettler et al., 2022), which is also echoed in our results. Personality traits that were important predictors in the final step were Honesty-Humility and Emotionality. Since people with higher scores on Emotionality, or its equivalent from the five-factor model, Neuroticism, experience more fear of physical dangers and more anxiety in response to life stressors, it is not surprising that they also perceive COVID-19 as a more serious threat. People who score higher on the Honesty-Humility scale avoid manipulating others for personal gain, feel less tempted to break rules, and are uninterested in wealth and social status. Further analysis on the level of personality facets indicated that the Honesty-Humility facet Fairness was the most important predictor of COVID-19 threat beliefs. Since people with higher Fairness scores are unwilling to take advantage of other individuals or of society at large, it is possible that they are more likely to engage in preventative behaviors that protect not only themselves but also others, which in turn might affect their perception of COVID-19 threat. In addition, the Extraversion facet, Social boldness, was important for explaining individual differences in COVID-19 threat beliefs. Our results indicate that those who feel confident in a variety of social situations take COVID-19 less seriously, possibly because higher threat perception would cause dissonance with their affinity toward social situations.

In line with previous studies, we have found that the overall trust in scientists (Bicchieri et al., 2021; Sturgis et al., 2021) and the overall trust in the political system (Bruine de Bruin et al., 2020; Schaeffer, 2021) predict COVID-19-related beliefs. In contrast to studies finding that COVID-19 threat beliefs were related to trust in the health system (Udow-Phillips and Lantz, 2020; Antinyan et al., 2021), we did not find evidence for this relationship. However, we found a reliable relationship between beliefs related to vaccination and beliefs related to COVID-19, suggesting that more specific beliefs about the usefulness of health interventions that are also relevant for COVID-19 are better predictors than the generalized trust in the overall health system. Our set of predictors accounted for a large amount of variance in COVID-19 threat beliefs, with other related beliefs being the most important predictors after controlling for individual differences in demographic characteristics and personality traits.

Our findings can be useful for planning interventions to target specific COVID-19 threat beliefs. Specifically, we found that people who do not perceive COVID-19 as a serious threat in Croatia tend to be younger and do not have a chronic disease, live in the southern region, are inclined to break rules for personal profit, do not worry in stressful situations, and do not trust vaccines, scientists, or the political system. It is interesting to note that the most recent incidence of COVID-19 is the highest in five counties in southern Croatia (Croatian Bureau of Public Health, 2021), suggesting that the COVID-19 threat beliefs we measured are valid indicators of a higher future incidence of COVID-19.

One of the limitations of this study is that it did not include beliefs in conspiracy theories, which have been shown to have a reliable effect on COVID-19 threat beliefs (Pavela Banai et al., 2021). Furthermore, we measured generalized beliefs about risks related to COVID-19, rather than more specific risk perceptions about the likelihoods of different outcomes one could experience. Further research could explore how these risk perceptions, which have been shown to influence willingness to engage in preventative behaviors (Dryhurst et al., 2020), relate to the three layers of individual differences we studied. In addition, this study did not include objective measures of behavioral outcomes. A possible further measurement issue is socially desirable responding, which might have influenced both COVID-19 threat beliefs, as well as one of the significant predictors, i.e., Honesty-Humility. However, a few studies examining socially desirable responding have indicated that the effects of social desirability are small, both for COVID-19 related issues, such as social distancing (Jensen, 2020) and Honesty-Humility (Müller and Moshagen, 2019). Finally, the study has been conducted in a specific context of the Croatian COVID-19 situation, which is both epidemiologically and culturally different from other countries. Cultural context can play a role in COVID-19 perceptions and consequences, especially when it comes to mental health (Konstantinov et al., 2020; Wang et al., 2020).

## Conclusion

Our study contributes knowledge about the joint influence of three different types of individual differences, namely, demographic characteristics, personality traits, and related beliefs in explaining differences in COVID-19 threat beliefs. Stable characteristics such as demographic characteristics and personality traits account for part of the individual differences in COVID-19 threat beliefs, but contextually, closer-related beliefs are the most important predictors of COVID-19 threat beliefs. Our results indicate that measuring personality at the facet level and the use of the HEXACO model could contribute to a better understanding of the individual differences in COVID-19 threat beliefs. While our study was not designed to explore causal links between COVID-19 threat beliefs and related beliefs, these results suggest that effective educational interventions should focus not only on the facts directly related to COVID-19 but also aim to strengthen the overall trust of people on scientists, political system, and vaccination in general.

## Data Availability Statement

The raw data supporting the conclusions of this article will be made available by the authors, without undue reservation.

## Ethics Statement

This study was reviewed and approved by the Ethics Committee of the Department of Psychology, University of Zagreb. The participants provided their written informed consent to participate in this study.

## Author Contributions

Both authors contributed to all aspects of the manuscript.

## Conflict of Interest

The authors declare that the research was conducted in the absence of any commercial or financial relationships that could be construed as a potential conflict of interest.

## Publisher’s Note

All claims expressed in this article are solely those of the authors and do not necessarily represent those of their affiliated organizations, or those of the publisher, the editors and the reviewers. Any product that may be evaluated in this article, or claim that may be made by its manufacturer, is not guaranteed or endorsed by the publisher.

## Footnotes

1
www.unipark.de
